# Epidithiodiketopiperazines (ETPs) exhibit in vitro antiangiogenic and in vivo antitumor activity by disrupting the HIF-1α/p300 complex in a preclinical model of prostate cancer

**DOI:** 10.1186/1476-4598-13-91

**Published:** 2014-04-28

**Authors:** Kelie M Reece, Emily D Richardson, Kristina M Cook, Tessa J Campbell, Stephen T Pisle, Alesia J Holly, David J Venzon, David J Liewehr, Cindy H Chau, Douglas K Price, William D Figg

**Affiliations:** 1 Molecular Pharmacology Section, Center for Cancer Research, National Cancer Institute, Bethesda, MD 20892, USA; 2 Clinical Pharmacology Core, Center for Cancer Research, National Cancer Institute, Bethesda, MD 20892, USA; 3 Biostatistics & Data Management Section, Center for Cancer Research, National Cancer Institute, Bethesda, MD 20892, USA; 4 Clinical Pharmacology Program, SAIC-Frederick, Frederick National Laboratory for Cancer Research, Frederick, MD 21702, USA; 5 NIH/NCI, Bldg. 10/Room 5A01, 10 Center Drive, 9000 Rockville Pike, Bethesda, MD 20892, USA

**Keywords:** Hypoxia, HIF-1α, p300, ETPs, Angiogenesis, VEGF

## Abstract

The downstream targets of hypoxia inducible factor-1 alpha (HIF-1α) play an important role in tumor progression and angiogenesis. Therefore, inhibition of HIF-mediated transcription has potential in the treatment of cancer. One attractive strategy for inhibiting HIF activity is the disruption of the HIF-1α/p300 complex, as p300 is a crucial coactivator of hypoxia-inducible transcription. Several members of the epidithiodiketopiperazine (ETP) family of natural products have been shown to disrupt the HIF-1α/p300 complex *in vitro*; namely, gliotoxin, chaetocin, and chetomin. Here, we further characterized the molecular mechanisms underlying the antiangiogenic and antitumor effects of these ETPs using a preclinical model of prostate cancer. In the rat aortic ring angiogenesis assay, gliotoxin, chaetocin, and chetomin significantly inhibited microvessel outgrowth at a GI_50_ of 151, 8, and 20 nM, respectively. *In vitro* co-immunoprecipitation studies in prostate cancer cell extracts demonstrated that these compounds disrupted the HIF-1α/p300 complex. The downstream effects of inhibiting the HIF-1α/p300 interaction were evaluated by determining HIF-1α target gene expression at the mRNA and protein levels. Dose-dependent decreases in levels of secreted VEGF were detected by ELISA in the culture media of treated cells, and the subsequent downregulation of *VEGFA*, *LDHA*, and *ENO1* HIF-1α target genes were confirmed by semi-quantitative real-time PCR. Finally, treatment with ETPs in mice bearing prostate tumor xenografts resulted in significant inhibition of tumor growth. These results suggest that directly targeting the HIF-1α/p300 complex with ETPs may be an effective approach for inhibiting angiogenesis and tumor growth.

## Introduction

Hypoxia, a reduction in tissue oxygen levels below physiological levels, is a nearly universal hallmark of solid tumors, and commonly develops due to heterogeneous blood flow from structurally and functionally abnormal blood vessels within the tumor [1,2]. Intratumoral hypoxia can drive tumor progression leading to a malignant phenotype that is associated with increased risk of invasion, metastasis, treatment failure, and patient mortality. Adaptation to hypoxia is critical for tumor survival and growth and is mediated through activation of heterodimeric hypoxia inducible factor 1 (HIF-1), which facilitates transcriptional responses to changes in oxygen levels [3-7].

HIF-1 is a heterodimeric transcription factor composed of an O_2_-regulated HIF-1α subunit and a constitutively expressed HIF-1β subunit. Under oxygenated conditions, HIF-1α is rapidly degraded via the von Hippel-Lindau tumor suppressor gene product (pVHL)-mediated ubiquitin-proteasome pathway. However, in hypoxic conditions, HIF-1α is stabilized and translocates into the nucleus where it binds as a heterodimer with HIF-1β to its cognate DNA sequence, the hypoxia response element (HRE). The heterodimer then recruits the p300/CBP family of coactivators to initiate the transcription of a diverse group of genes involved in the adaptive response to hypoxia [3,5,7,8].

HIF-1 expression is elevated in many human cancers, and its levels in cells correlate with tumorigenicity and angiogenesis, the formation of new blood vessels via the sprouting and remodeling of preexisting vessels [3-5,9,10]. The newly generated blood vessels are required to supply adequate oxygen and nutrition to the growing tumor mass, which further accelerates tumor growth and facilitates metastasis. Thus, tumor angiogenesis plays a key role in cancer cell survival, tumor growth and metastasis [1,11-13]. In particular, HIF plays a central role in the activation of multiple genes that encode angiogenic growth factors, including VEGF, the most potent stimulator of angiogenesis, stromal-derived factor 1 (*SDF1*), placental growth factor (*PGF*), and angiopoietin (*ANGPT*) 1 and 2 [14,15]. Therefore, inhibition of HIF-1 is an attractive therapeutic strategy for targeting hypoxia and tumor angiogenesis.

Many HIF-1 inhibitors have been generated in the last several years, most of which function by altering signal transduction pathways that are indirectly associated with HIF or that are part of more complex pathways relevant to human cancer, clearly limiting their specificity of action and increasing their likelihood of toxicity [10,16,17]. As a result, most of these inhibitors have failed in clinical trials. Thus, there is a clear need for direct HIF inhibitors, yet no such agent has been clinically developed to date [10,16]. One promising approach for directly inhibiting HIF is by disrupting the complex that HIF-1α forms with p300, an essential transcriptional coactivator [10,18]. Previous research from our laboratory showed that several members of the epidithiodiketopiperazine (ETP) family of fungal secondary metabolites; namely, gliotoxin, chaetocin, and chetomin are able to block the interaction between HIF-1α and p300 *in vitro* by a zinc ejection mechanism [19,20].

Angiogenesis plays a critical role in prostate cancer development and progression, and inhibition of angiogenesis in preclinical models has been shown to be an effective target in metastatic prostate cancer. Thus, in this study, we used prostate cancer cells as a preclinical model to further characterize the molecular mechanisms of these compounds in respect to their antiangiogenic effects. Data from rat aortic ring assays demonstrated the antiangiogenic properties of these ETPs, and co-immunoprecipitation experiments showed that these effects are due, at least in part, to disruption of the HIF-1α/p300 complex, which led to a subsequent decrease in HIF activity. We also demonstrated that these ETPs have antitumor efficacy *in vivo*. Taken together, these data indicate that directly inhibiting HIF-1 by targeting the HIF-1α/p300 interaction with these ETPs may prove valuable in suppressing tumor angiogenesis and prostate cancer progression.

## Materials and methods

### Cell culture

PC3 human prostate cancer cells (ATCC; Manassas, VA) were grown in Ham’s F-12K medium (Gibco; Carlsbad, CA), and HCT116 human colon cancer cells (ATCC) were grown in McCoy’s 5a medium (Gibco). Both cell lines were supplemented with 10% fetal bovine serum (Atlanta Biologicals; Lawrenceville, GA), 50 U/ml penicillin, and 50 mg/ml streptomycin (Gibco). Cells were grown in a 37°C incubator with 5% CO_2_. For hypoxic conditions, 200 μM of cobalt chloride (CoCl_2_) was added to the media, or cells were placed in a modular incubator chamber flushed with 1% O_2_, 5% CO_2_, 94% N_2_.

### Antibodies and reagents

Monoclonal HIF-1α antibody was purchased from BD Biosciences Pharmingen (San Diego, CA). Monoclonal p300 antibodies were purchased from Thermo Scientific (Rockford, IL) and Calbiochem (Billerica, MA). Alexa Fluor 680 goat anti-mouse IgG for fluorescence detection using the Odyssey Imaging System was from Molecular Probes (Eugene, OR). The FITC rabbit anti-mouse secondary antibody was used for immunofluorescence staining, and was purchased from Abcam (Cambridge, MA). Protein A/G Agarose was purchased from Thermo Scientific, and CoCl_2_ was purchased from Sigma (St. Louis, MO). Gliotoxin, chaetocin, and chetomin were purchased from Sigma, and sorafenib was purchased from Toronto Research Chemicals (Ontario, Canada). Each of the ETPs and sorafenib were stored frozen in DMSO. Odyssey blocking buffer was from LICOR (Lincoln, NE).

### Rat aortic ring assay

The rat ring assay was performed similarly to that previously described [21]. Twelve-well tissue culture plates were covered with 250 μl Matrigel (BD Biosciences) and allowed to gel for 30 to 45 min at 37°C, 5% CO_2_. Thoracic aortas were excised from 6- to 8-week-old male Sprague–Dawley rats, and the fibroadipose tissue was removed. The aortas were cut into 1 mm-long cross-sections and placed on the Matrigel-coated wells. They were then covered with an additional 250 μl Matrigel and allowed to gel for 30 to 45 min at 37°C. The rings were cultured for 24 h in 1 ml EGM-2 (Atlanta Biologicals). After 24 h, the medium was removed and replaced with 1 ml EBM-2 (Atlanta Biologicals), supplemented with fetal bovine serum (2%), ascorbic acid, hydrocortisone, heparin, and amphotericin, at concentrations consistent with EGM-2 media. Each ETP (gliotoxin, chaetocin, chetomin) was dissolved in DMSO and added to the EBM media (final DMSO concentration of 0.1%) before being added to the well. The rings were incubated in treatment media for 5 days, after which they were imaged on an inverted phase contrast microscope. All images were acquired with Spot version 4.5 imaging software. Vascular outgrowth was quantified using Adobe Photoshop CS3 version 10; the inner portion of the rings was excluded from analysis.

### Immunofluorescence

Rat ring assays were performed as described in the previous section; however, 6-well tissue culture plates were used and no drug was added. The rings were incubated for 7 days in EGM-2 media, after which the media was removed and 2 ml of Cell Recovery Solution was added to the wells (BD Biosciences) to depolymerize the Matrigel and release the endothelial cells. After a 2-h incubation, the recovery solution was removed, the cells were washed once in PBS containing 0.1% Tween (PBS-Tween), and were then fixed in 4% paraformaldehyde for 30 min at 37°C. Subsequently, the cells were washed again with PBS-Tween and permeabilized with 0.5% Triton X-100 (Sigma) for 4 min. After another PBS-Tween wash, the cells were blocked for 1 h in PBS containing 2% BSA (Sigma), and then incubated for 1 h in a 1:50 dilution of HIF-1α antibody. After three washes with PBS-Tween, the rings were incubated for 1 h in the dark with a 1:500 dilution of FITC-labeled anti-mouse antibody. The cells were washed three times with PBS-Tween and mounted onto slides with VECTASHIELD Mounting Medium with DAPI (Vector Laboratories; Burlingame, CA). Images were taken on an Olympus BX51 microscope with UplanF1 40x lens. InSight Firewire camera and Spot version 4.5 imaging software were used to capture the images. Adobe Photoshop CS3 version 10 was used for after-capture edits where all photos were processed the same and merged equally.

### HIF-1α/p300 co-immunoprecipitation

PC3 cells were treated with media containing 200 μM CoCl_2_ and either DMSO or the indicated concentrations of ETPs. The media covering the cells was removed after 18 h, and the cells were washed once with PBS containing CoCl_2_. Cells were then scraped from the plates in 200 μl of ice-cold lysis buffer containing 25 mM Tris, pH 8.0, 150 mM NaCl, 2 mM EDTA, 1% Triton, and protease and phosphatase inhibitors (Nacalai; San Diego, CA). The lysed cells were pipetted into a 1.5-ml Eppendorf tube, incubated on ice for 30 min, and microcentrifuged at 14,000 × *g* for 30 min at 4°C. Clarified lysates were incubated overnight at 4°C with 0.3 μg of p300 monoclonal antibody (Calbiochem), and then incubated for 1 h with Protein A/G Agarose. Beads were extensively washed in lysis buffer, and bound proteins were eluted in SDS sample buffer and subjected to Western blot analysis.

### Western blot analysis

SDS-solubilized protein samples were resolved using the Novex NuPage SDS-PAGE gel system (Invitrogen; 3-10% Tris Acetate gels for p300 detection, 4-12% Bis-Tris gels for HIF-1α detection), and electrophoretically transferred to 0.45 μm nylon-supported nitrocellulose membranes (Biorad; Hercules, CA). Membranes were blocked for 1 h in Odyssey blocking buffer, and then incubated overnight at 4°C in a 1:1000 dilution of HIF-1α monoclonal antibody (BD Biosciences) and a 1:500 dilution of p300 monoclonal antibody (Thermo Scientific). After three washes in lysis buffer for 5 min each, the membranes were incubated for 1 h at room temperature in a 1:10,000 dilution of fluorophore-conjugated goat anti-mouse IgG, and washed another three times for 10 min each. Bound antibodies were visualized via the Odyssey Infrared Imaging System and Odyssey software.

### Cell viability assays

HCT116 and PC3 cells were seeded overnight into 96-well plates in 100 μl of medium at a concentration of 5 × 10^4^ cells well^−1^. After overnight incubation at 37°C, medium was removed and replaced with 200 μl of medium containing increasing concentrations of ETPs or vehicle control (DMSO). Plates were placed in either a normoxic incubator or a hypoxic chamber (Billups-Rothenberg; Del Mar, CA) for 18 h. Cell viability was measured by adding 20 μl CellTiter-Blue cell viability reagent (Promega; Madison, WI) to each well, after which the cells were returned to the 37°C incubator until sufficient color change. Fluorescence intensity was read at 570 nm using a SpectraMax M2 fluorescence plate reader (Molecular Devices; Sunnyvale, CA).

### VEGF ELISA

HCT116 and PC3 cells were seeded into 96-well plates at a concentration of 50,000 cells/ml and 190,000 cells/ml, respectively. After overnight incubation at 37°C, the media was removed and replaced with 210 μl serum-free media containing either drug or vehicle control (DMSO), in the absence or presence of 200 μM cobalt chloride. The plates were incubated for 18 h at 37°C. The supernatant was then collected on ice, after which the number of viable cells in each well was determined using the CCK8 assay (Dojindo Molecular Technologies; Rockville, MD). After cell viability assessment, the concentration of secreted VEGF in the tissue culture supernatant was determined using the Quantikine human VEGF ELISA Kit (R & D Biosystems; Minneapolis, MN) according to the manufacturer’s instructions. Relative VEGF concentrations in the supernatant were normalized to the cell number in each well.

### Semi-quantitative real time-PCR (qPCR)

HCT116 and PC3 cells were treated for 18 h with ETPs under hypoxic conditions (hypoxic chamber or treatment with 200 μM CoCl_2_). Total RNA extraction was performed using the RNAeasy mini kit (Qiagen; Valencia, CA) according to the manufacturer’s protocol. RNA concentration was determined using a NanoDrop® spectrophotometer (Molecular Devices). Purified RNA (1.5 μg) from HCT116 cells was reverse transcribed per 25-μl cDNA synthesis reaction using the RT^2^ First Strand kit (SABiosciences; Valencia, CA) according to the manufacturer’s instructions. Purified RNA (0.24-0.32 μg) from PC3 cells was reverse transcribed per 20 μl cDNA synthesis reaction using The Superscript III First-Strand Synthesis System for RT-PCR (Invitrogen) according to the manufacturer’s protocol.

For qPCR reactions with RNA extracted from HCT116 cells, cDNA reaction products (25 μl) were diluted 1:4 in water. For each sample, 1 μl each of cDNA, forward and reverse primers (VEGFA, ENO1, LDHA, ACTB), were mixed with 2x RT^2^ SYBR Green/ROX qPCR Master Mix (SABiosciences) in a total volume of 25 μl. qPCR was performed using a Stratagene Mx3005P™ Real-Time PCR System with MxPro analysis software (Stratagene; Santa Clara, CA). For qPCR reactions with RNA extracted from PC3 cells, cDNA reaction products (20 μl) were diluted 1:3 in water. For each sample, 2 μl of cDNA was mixed with 18 μl master mix containing 1 μl of forward and reverse primers, 7 μl water, and 10 μl Taqman Gene Expression Master Mix (Applied Biosystems) for a total volume of 20 μl. qPCR was performed using an Applied Biosystems StepOnePlus Real-Time PCR system with StepOne Software. All qPCR reactions were run in triplicate. β-Actin (ACTB) was used as a reference housekeeping gene. Fold-change in RNA levels was calculated using the ΔΔC_t_ method (SABiosciences 2009 RT2 Profiler PCR Array System User Manual; Frederick, MD).

### Xenografts

Male, Fox Chase SCID Beige Mice were purchased at 5 weeks of age from the National Cancer Institute Animal Production Area (Frederick, MD). Animals were housed in polycarbonate cages, and kept on a 12-h light/dark cycle with food and water given ad libitum. The National Cancer Institute is accredited by AAALAC International and follows the Public Health Service Policy for the Care and Use of Laboratory Animals. Animal care was provided in accordance with the procedures outlined in the “Guide for Care and Use of Laboratory Animals” (National Research Council; 1996; National Academy Press; Washington, DC). The study protocol was approved by the NCI Animal Care and Use Committee (Bethesda, MD).

Xenografts were generated by subcutaneously injescting mice with 3 million PC3 or DU-145 cells suspended in 100 μl of sterile PBS. Tumor volume (V) was calculated according to the following formula: V = length  × width  ×  heigth  ×  0.52. When tumor volume reached 100 mm^3^, mice were divided randomly into treatment and control groups of 7 rats each. ETPs and vehicle (12.5% DMSO, 37.5% PEG 400, 50% sterile saline) were administered to the animals by daily intraperitoneal injection at the previously determined MTD doses of 0.50 mg/kg, 0.25 mg/kg, and 0.50 mg/kg for gliotoxin, chaetocin, and chetomin, respectively, at a dosing volume of 5 ml/kg. Tumors were measured 3 times a week. After 2 weeks, tumors were excised and frozen for subsequent immunohistochemistry analysis.

### Immunohistochemistry

Frozen samples of tumor xenografts were submitted to the Pathology/Histotechnology Laboratory (PHL) at NCI-Frederick. Samples were thawed in 10% buffered neutral formalin, paraffin-embedded, and cut into 5-mm sections. Staining was performed using the Bond Max Autostainer from Leica Biosystems (Buffalo Grove, IL). EDTA was used for antigen retrieval and 10% normal rabbit serum (Vector Laboratories) was used as blocking buffer. Sections were incubated with a 1:100 dilution of VEGF or CD31 antibodies (R & D Systems) for 30 min, followed by incubation with a 1:500 dilution of biotinylated secondary antibody (Vector Laboratories) for 30 min. Immune reactivity was visualized with the Intense R Detection Kit (Leica Biosystems). Sections were counterstained with hematoxylin and eosin (H & E). All slides were imaged on an Aperio bright field ScanScope.

### Statistical analysis

Analysis of variance (ANOVA) was performed on either raw or transformed data. Various fixed effects models, mixed models, or repeated measures ANOVA were used as appropriate. Hochberg’s method was used for adjusting p-values for multiple comparisons. For the xenograft model, treatment groups were compared using Mann–Whitney *U* test. P values less than 0.05 were considered statistically significant, and all reported p-values are two-tailed.

## Results

### ETPs decrease microvessel outgrowth from rat aortic rings

**Figure 1:**
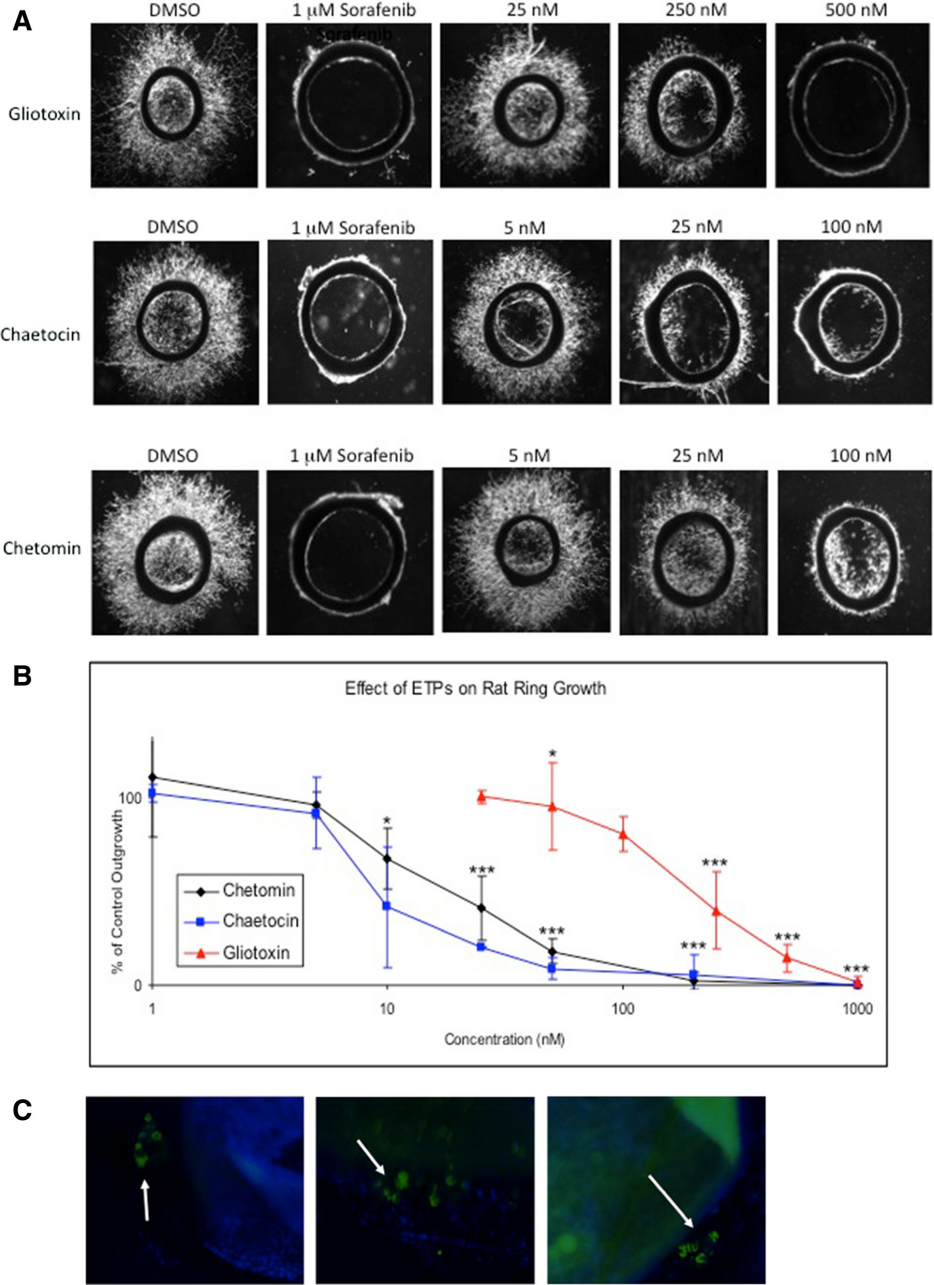
**Rat ring assay shows dose-dependent inhibition of microvessel outgrowth by ETPs. A***,* Cells were treated with DMSO (negative control), 1 μM sorafenib (positive control), and increasing concentrations of gliotoxin, chaetocin, and chetomin. Three concentrations of each ETP are shown to illustrate the dose-dependent effect of the drugs on microvessel outgrowth. **B***,* Dose response curves showing a decrease in outgrowth with increasing ETP concentrations. Gliotoxin, chaetocin, and chetomin had a GI_50_ of 151, 8, and 20 nM, respectively. Data points are presented as mean S.E.M (*error bars*) from independent experiments run in triplicate (*n* = 6). *, *p* < 0.05, **, *p* < 0.001, ***, *p* < 0.0001. **C***,* Rat aortic rings at day 5 were stained with a HIF-1α monoclonal primary antibody and a FITC-conjugated secondary antibody to label endothelial cells. The merged image shows DAPI-stained nuclei in blue and HIF-1α expression in green (indicated by arrows). Three representative images are shown.

To determine if gliotoxin, chaetocin, and chetomin have antiangiogenic properties, a series of rat aortic ring assays were performed with increasing concentrations of each ETP. Specifically, ETPs were added to the culture, and their effect on vessel outgrowth was determined using image analysis software to measure the length and abundance of the sprouting microvessels. DMSO was used as a negative control and sorafenib, a known antiangiogenic agent used to treat hepatocellular carcinoma and kidney cancer [17], was used as a positive control. As seen in Figure 1A, a dose-dependent decrease in microvessel outgrowth occurred with increasing concentrations of each ETP. Quantification of vessel outgrowth, where mean percent inhibition of vessel growth was compared to vehicle, confirmed the dose-dependent effect of the ETPs (Figure 1B). At a concentration of 25 nM, gliotoxin exhibited little to no effect on outgrowth, whereas inhibition was statistically evident at concentrations of 250 nM and above; 500 nM of gliotoxin led to greater than 90% inhibition (*p* < 0.0001). At concentrations of 1 and 5 nM, chaetocin and chetomin had little effects on outgrowth, but 25 nM and above led to inhibition of angiogenesis; 100 nM of either ETP inhibited approximately 90% of microvessel outgrowth (*p* < 0.0001). The GI_50_ of gliotoxin, chaetocin, and chetomin was 151, 8, and 20 nM, respectively.

A previous *in vitro* study from our laboratory used a fluorescent binding assay to show that ETPs are capable of disrupting the interaction between the C-TAD domain of HIF-1α and the CH1 domain of p300, and electrospray ionization mass spectrometry (ESI-MS) analyses determined that this occurs via a zinc ejection mechanism [19,22,23]. Since the interaction between HIF-1α and p300 is critical for transactivation, blocking this complex reduces HIF-1α-induced transcription of angiogenic genes [23-25]. Thus, it seemed likely that the antiangiogenic effects of the ETPs observed in the rat aortic ring assays were mediated through disruption of the HIF-1α/p300 complex. To test this hypothesis, we first immunostained the aortic rings for HIF-1α to confirm its presence in the endothelial cells of the microvessel outgrowth (Figure 1C).

### ETPs disrupt the interaction of HIF-1α and p300 in cells

**Figure 2:**
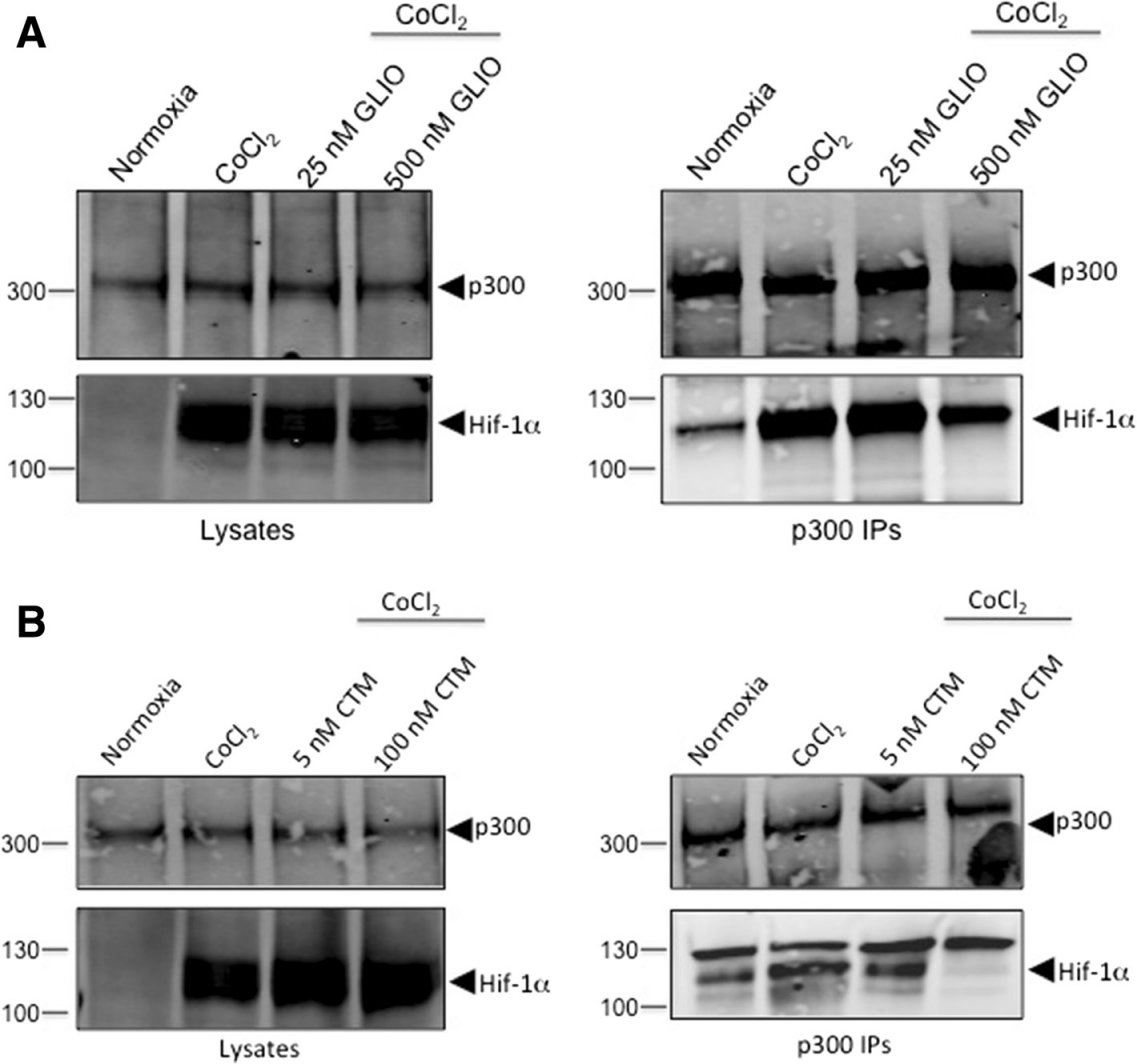
**ETPs disrupt the endogenous HIF-1α/p300 complex in cells.** PC3 cells were cultured under normoxia or hypoxia (CoCl_2_) in the presence or absence of the indicated concentrations of gliotoxin and chaetocin. After 18 h, co-immunoprecipitations were performed from the cell lysates using a p300 mouse monoclonal antibody and Protein A/G Agarose. Bound proteins were eluted with SDS buffer followed by immunoblot analysis of the lysates (*left panels*) and immune complexes (*right panels*) with antibodies recognizing p300 and HIF-1α. **A**, Treatment with 25 nM and 500 nM gliotoxin (GLIO). **B**, Treatment with 5 nM and 100 nM chaetocin (CTM) (*n* = 3 for all).

Next, we performed co-immunoprecipitation experiments to determine if the ETPs could disrupt the interaction of endogenous HIF-1α and p300. Briefly, endogenous p300 was immunoprecipitated from PC3 human prostate cancer cells under hypoxic conditions in the absence and presence of ETPs, and co-immunoprecipitated HIF-1α was detected by Western blotting. The low and high concentrations of ETPs used in this experiment represented those concentrations that either had little effect on angiogenesis or that inhibited approximately 90% of microvessel outgrowth in the rat ring assay, respectively. As expected, the expression levels of p300 in the cell lysates remained the same under both normoxia and hypoxia (200 μM CoCl_2_; a hypoxia mimetic), and no HIF-1α expression could be seen under normoxic conditions (Figure 2A, B; *left panel*). Western blot analysis of the immune complexes showed no effect in the presence of 25 nM gliotoxin; however, in the presence of 500 nM gliotoxin, less HIF-1α co-immunoprecipitated with p300 (Figure 2A; *right panel*). Similarly, treatment of cells with only 5 nM chetomin did not cause disruption of the complex, but 100 nM chetomin was able to disrupt the HIF-1α/p300 complex (Figure 2B; *right panel*). Neither gliotoxin nor chetomin affected the expression levels of HIF-1α or p300 under hypoxic conditions. Thus, the observed decrease in the amount of HIF-1α in the p300 immunoprecipitates upon treatment with gliotoxin and chetomin demonstrates their ability to disrupt the interaction between HIF-1α and p300 in cells. In accordance with a previous study [26], chaetocin treatment inhibited HIF-1α expression in a dose-dependent manner (data not shown); thus, co-immunoprecipitations could not be performed from chaetocin-treated cells.

### ETPs decrease VEGF secretion in a dose-dependent manner

**Figure 3:**
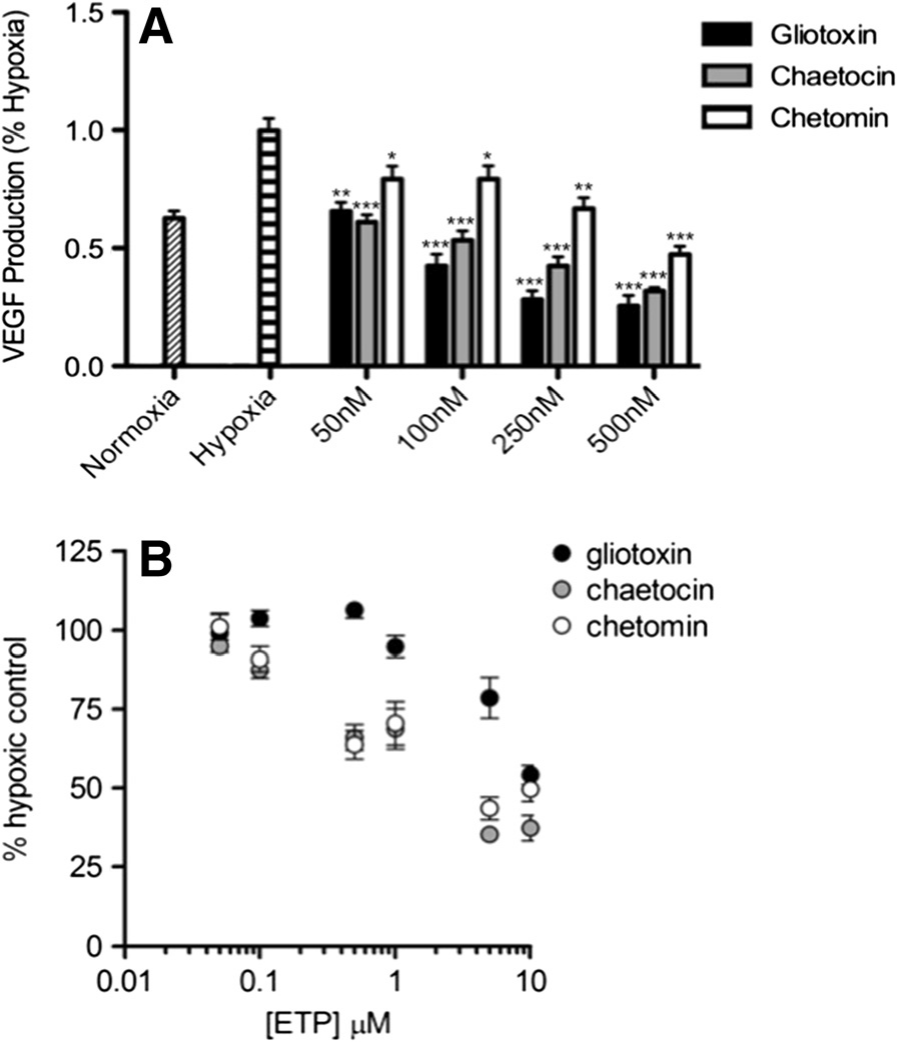
**ETPs decrease VEGF secretion in a dose-dependent manner. A**, Hypoxia was induced for 18 h in PC3 cells in the absence or presence of the indicated concentrations (1000 nM is not shown) of gliotoxin, chaetocin, and chetomin. This was followed by ELISA quantification of secreted VEGF normalized to DMSO under hypoxic conditions. A repeated measures ANOVA was performed on the data; Hochberg’s post-hoc method was used to adjust the *p*-values. Data are presented as mean ± S.E.M from independent experiments run in triplicate (*n* = 2-7). *, *p* < 0.05, **, *p* < 0.001, ***, *p* < 0.0001. **B**, PC3 cells were treated with increasing concentrations of ETPs or with vehicle control (DMSO). Plates were placed in either a normoxic incubator or hypoxic chamber for 18 h. Cell viability was then determined using the CellTiter-Blue cell viability reagent. Data points are presented as mean ± S.E.M from independent experiments run in triplicate (*n* = 4).

To determine whether blocking the HIF-1α/p300 complex in cells with ETPs affected angiogenic signaling, VEGF secretion was examined in the presence of ETPs since VEGF is a prominent target of HIF-1α and a potent stimulator of angiogenesis. To this end, PC3 cells were treated with different concentrations of each ETP under hypoxic conditions. After 18 hours of treatment, cell culture media was collected, and a VEGF ELISA was performed to measure the effects of ETPs on VEGF levels in the media. As shown in Figure 3A, treatment with gliotoxin, chaetocin, and chetomin led to a dose-dependent decrease in hypoxia-induced VEGF expression. This trend was also seen in HCT116 colorectal carcinoma cells (Additional file 1: Figure S1A), indicating that the observed effects with ETPs were not limited to PC3 cells. Interestingly, gliotoxin did not have an effect on VEGF expression in this cell line (at the concentrations shown), signifying that this compound may have cell-specific effects.

To ensure that the observed decrease in secreted VEGF levels was not due to a nonspecific reduction in cell viability, PC3 cells were treated with increasing concentrations of gliotoxin, chaetocin, and chetomin under hypoxic conditions, followed by measurement of cell viability. Significant toxicity was not observed, as only concentrations close to 10 μM decreased cell viability to approximately 50% in both cell lines (Figure 3B), whereas nanomolar concentrations of ETPs, which did not impact cell viability, were able to significantly reduce VEGF levels (Figure 3A). Similar results were obtained for HCT116 cells up to 1 µM (Additional file 1: Figure S1B). Together with our previous data, these results demonstrate that the potent effect of these ETPs in reducing VEGF protein levels was not caused by general cellular cytotoxicity.

### ETPs inhibit HIF-1α transcriptional activity

**Figure 4:**
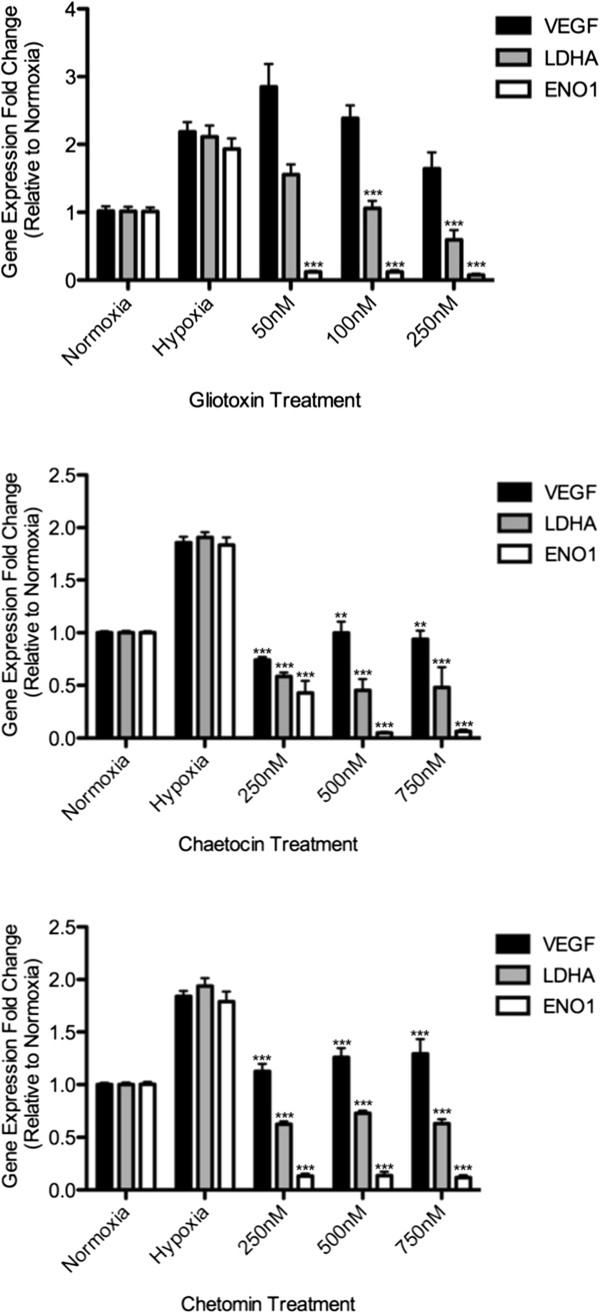
**ETPs decrease expression of HIF-1α-dependent target genes.** PC3 cells were seeded into 6-well plates and incubated for 18 h under normoxic or hypoxic conditions, in the absence or presence of the indicated concentrations of gliotoxin (*top panel*), chaetocin (*middle panel*), or chetomin (*bottom panel*). Total RNA was harvested and tested for *VEGF*, *LDHA*, and *ENO1* mRNA expression by qPCR, as described in the Materials and methods. Results are expressed as fold increase relative to mRNA levels under hypoxic conditions in the absence of ETPs. β-actin was tested in parallel as an internal control for input RNA. Results are the mean ± S.E.M of independent experiments run in triplicate (*n* = 2-5). A repeated measures ANOVA was performed on the data with Hochberg’s post-hoc method. *, *p* < 0.05, **, *p* < 0.001, ***, *p* < 0.0001.

Since HIF-1α is a transcription factor, it was important to determine if disrupting the HIF-1α/p300 complex specifically affected HIF-1α transcriptional activity. Since there are several pathways and processes that regulate the production of VEGF protein [27,28], we could not definitively ascertain that the observed decrease in secreted VEGF was due to a decrease in HIF-1α activity. Therefore, as a way to measure HIF-1α transcriptional activity, we performed qPCR in the absence and presence of ETPs and monitored levels of *VEGF* mRNA. As an additional way of demonstrating the specificity of HIF-1α transcriptional activation, we also monitored the levels of two other HIF-1α target genes; namely lactate dehydrogenase A (*LDHA*) and enolase-1 (*ENO1*), which have one hypoxia response element (HRE) and three HRE sites, respectively that are required for expression [29]. Briefly, PC3 cells were incubated under normoxic or hypoxic conditions in the absence or presence of the indicated concentrations of gliotoxin, chaetocin, and chetomin. After 18 hours, total cellular RNA was extracted from the cells, followed by qPCR; β-actin was used as a reference housekeeping gene. As shown in Figure 4, levels of *VEGF*, *LDHA*, and *ENO1* were lower in normoxia, and increased about 2-fold under hypoxic conditions, which was expected since HIF-1α transcriptional activity increases under hypoxic conditions due to a lack of prolyl hydroxylases, which cause HIF-1α degradation under normoxia. There was a significant decrease in *ENO1* levels at each gliotoxin concentration tested (*p* < 0.0001), and in *LDHA* concentrations of 100 nM and above (*p* < 0.0001). *VEGF* expression decreased with 250 nM gliotoxin but did not reach statistical significance (Figure 4; *top panel)*. This observed decrease in HIF-1α gene expression upon ETP treatment was also confirmed in HCT116 cells (Additional file 2: Figure S2; *top panel*), although similar to what we observed in the VEGF ELISA, gliotoxin treatment of HCT116 cells did not affect *VEGF* gene expression, nor were decreases observed in levels of *LDHA* or *ENO1*, indicating that gliotoxin does not target HIF-1α in this cell line. When PC3 cells were treated with chaetocin (Figure 4; *middle panel*) and chetomin (Figure 4; *bottom panel*) under hypoxic conditions, there was a significant decrease in *VEGF, LDHA*, and *ENO1* gene expression to approximately normoxic levels at each of the indicated concentrations (*p*<0.05). Similar effects of chaetocin (VEGF and LDHA only) and chetomin (VEGF only) were seen in HCT116 cells as well (Additional file 2: Figure S2; *middle and bottom panels*). Together, these data demonstrate that disrupting the HIF-1α/p300 complex by ETPs has functional consequences, as revealed by the inhibitory effect of ETPs on HIF-1α transactivation in both cell lines.

### ETPs inhibit xenograft tumor growth

**Figure 5:**
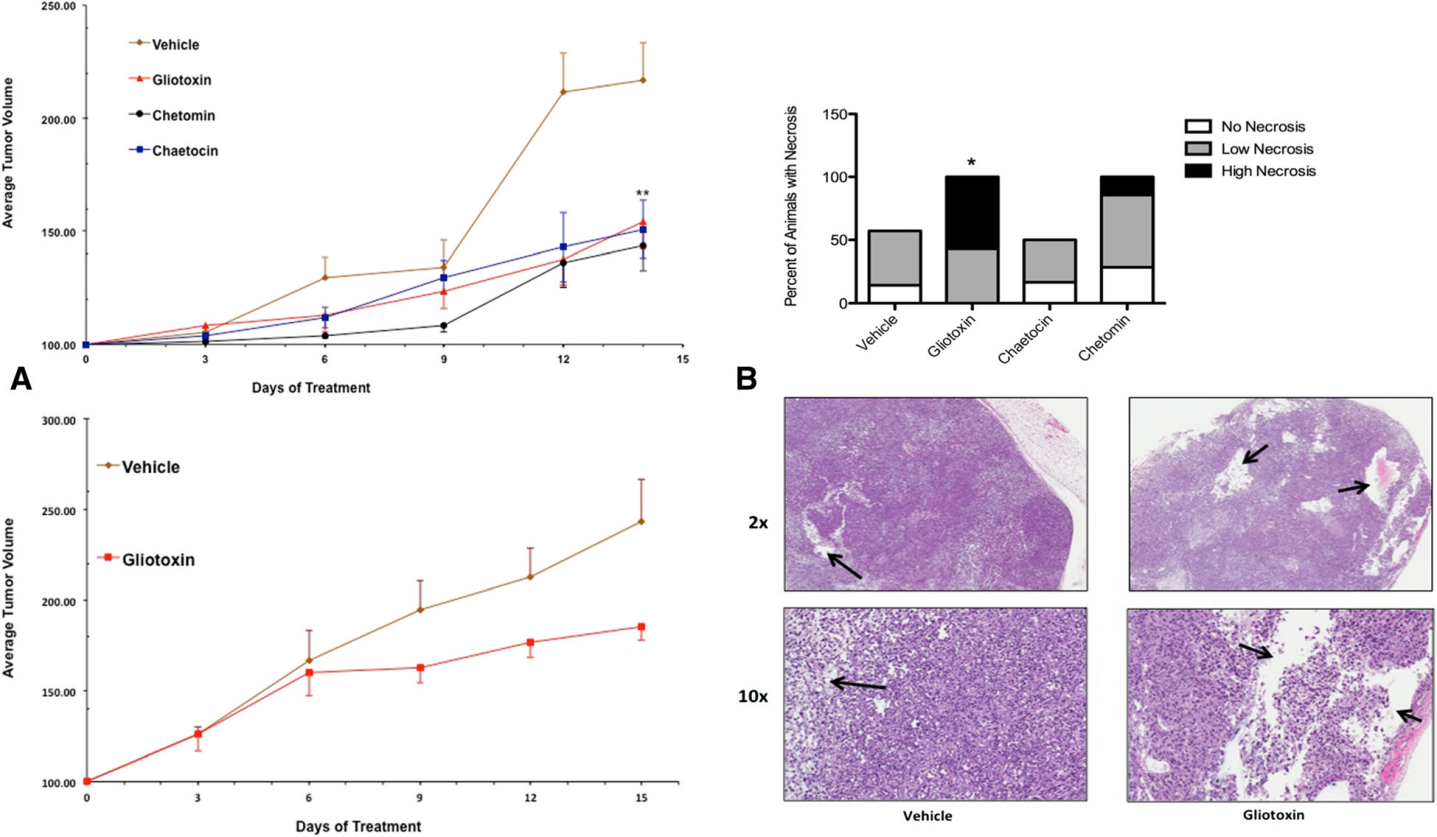
**ETPs attenuate tumor growth *in vivo*. A**, Mice with approximately 100 mm^3^ PC3 xenografts were treated by daily intraperitoneal injection for 15 days with gliotoxin (0.50 mg/kg), chaetocin (0.25 mg/kg), chetomin (0.50 mg/kg), or vehicle control (*top panel*). Mice with approximately 100 mm^3^ DU-145 xenografts were treated by daily intraperitoneal injection for 15 days with gliotoxin (0.50 mg/kg) or vehicle control (*bottom panel*). Results are the mean of 6–7 independent tumors ± S.E.M. **B**, Graphical representation of the percent of animals with varying degrees of necrosis (*top panel*). Statistical significance was determined using the Mann–Whitney U test. *, *p* < 0.05, **, *p* < 0.005. Low-power views (2x and 10x) of H & E-stained tumor sections from PC3 xenografts (*bottom panel*). The arrows indicate areas of necrosis in vehicle- and gliotoxin-treated tumors, which had the highest incidence of necrosis.

In order to evaluate whether disruption of the HIF-1α/p300 complex by ETPs affected the growth of human tumor xenografts, PC3 cells were implanted subcutaneously into severe combined immunodeficiency (SCID) mice. Once tumors had grown to approximately 100 mm^3^, vehicle or ETPs were administered for 15 days by daily intraperitoneal injection. As seen in Figure 5A (*top panel*), compared to mice treated with vehicle control, 0.50 mg/kg gliotoxin (*p* = 0.0037), 0.25 mg/kg chaetocin (*p* = 0.0020), and 0.50 mg/kg chetomin (*p* = 0.0037) had significant antitumor activity. Since all of the ETPs had similar antitumor activity, we chose just one (gliotoxin) to move forward with in another xenograft study using DU-145 human prostate cancer cells. Similar to the PC3 xenograft model, gliotoxin had significant antitumor activity in DU-145 xenografts (Figure 5A; *bottom panel*). Daily administration of ETPs did not cause major weight loss (data not shown). Finally, histological examination of tumor sections revealed significant necrosis in gliotoxin-treated tumors compared to mice that received chaetocin, chetomin, or vehicle (Figure 5B). Immunostaining for VEGF and CD31 did not reveal appreciable differences between treatment groups (data not shown), although the variability in staining made the data difficult to interpret. Similar immunostaining issues were seen in the study by Kung *et al.,* who noted that immunostaining for VEGF revealed vast regional variability in chetomin-treated tumors [23].

## Discussion

Here, we show that ETPs abrogate hypoxia-induced transcription by disrupting the HIF-1α and p300 interaction, with consequent antiangiogenic and antitumor effects. Angiogenesis, the recruitment of new blood vessels from the existing vasculature, is essential for the growth and metastasis of solid tumors, as tumors will not grow beyond 1 to 2 mm in diameter without an independent blood supply [30]. Thus, inhibition of angiogenesis has emerged as a promising strategy for cancer treatment. Since the progression of cancer leads to hypoxic conditions that stimulate angiogenesis, an important objective of anti-cancer therapy is the development of new drugs that suppress the hypoxic response in solid tumors. HIF-1 is the most important mediator of a cell’s response to hypoxia, as it regulates genes that enable the cell to survive in the hypoxic environment, including those involved in cell proliferation, metastasis, and glycolysis [6].

One promising approach for directly inhibiting HIF activity is by using small molecules to target the critical interaction between HIF-1α and its coactivator, p300. Previous research from our laboratory and others showed that select members of the ETP family of fungal metabolites were able to disrupt the interaction between HIF-1α and p300 using an *in vitro* cell-free system [19,20,23]; namely, gliotoxin, chaetocin, and chetomin, all of which have a wide range of biological activities, including antimicrobial, anti-inflammatory, and anticancer effects [23,31,32]. In the present study, we further characterized the molecular mechanisms of the antiangiogenic and antitumor effects of ETPs using an *ex vivo* angiogenesis assay, *in vitro* cellular assays, and an *in vivo* animal model of prostate cancer. We began our studies with the rat aortic ring assay, which is a useful assay for testing angiogenic factors or inhibitors in a controlled environment. This model integrates the advantages of both *in vivo* and *in vitro* systems, and is unique in that it recapitulates all of the key steps in the angiogenic process; namely, matrix degradation, migration, proliferation, and reorganization [33]. Our data showed that each ETP caused a dose-dependent decrease in microvessel outgrowth, indicating that they possessed antiangiogenic activities *in vitro*. This is in accordance with data from Lee *et al.*, which showed that gliotoxin significantly inhibited angiogenesis, as assessed by a tube formation assay in human umbilical vein endothelial cells (HUVECs) [34].

To determine if the mechanism underlying their activity was via disruption of the HIF-1α/p300 complex, co-immunoprecipitation experiments of the endogenous complex were carried out in PC3 cells. Treatment of prostate cancer cells with gliotoxin and chetomin blocked the ability of HIF-1α to interact with p300, which is in accordance with previous cellular studies that showed disruption of the HIF-1α/p300 complex in HepG2 liver hepatocellular cells with chetomin [23], as well as *in vitro* studies that used recombinant proteins to show that gliotoxin [19,22] and chetomin [19,22,23] disrupted the binding of HIF-1α and p300. While *in vitro* studies have also shown that chaetocin is able to disrupt HIF-1α and p300 binding [19,35], we could not demonstrate this in our co-immunoprecipitation studies because interestingly, treatment with chaetocin decreased HIF-1α protein expression. A similar effect was seen in a study by Lee *et al*. [26], which demonstrated that the hypoxic induction of HIF-1α was attenuated by chaetocin in human hepatoma cell lines. The authors later concluded that the antiangiogenic and anticancer effects of chaetocin against hepatoma were via deregulation of HIF-1α premessenger RNA splicing. Taken together, these findings suggest that ETPs exhibit more than one mechanism of action, and these effects may be cell-type specific.

Our ELISA and qPCR experiments demonstrated the ability of the ETPs to prevent VEGF production in PC3 cells under hypoxic conditions. Interestingly, in HCT116 cells, gliotoxin did not have an effect on VEGF expression or that of the other HIF-1α target genes analyzed, indicating that gliotoxin does not target HIF-1α in this cell line. Previous work has suggested that the antitumor activity of gliotoxin against breast cancer *in vivo* may be through its ability to inhibit farnesyltransferases [32]. Since inhibition of this enzyme was recently shown to reduce angiogenesis by interrupting endothelial cell migration [35], farnesyltransferase could be a target of gliotoxin in HCT116 cells.

Finally, we demonstrated that gliotoxin, chaetocin, and chetomin were able to significantly decrease tumor growth in a xenograft model of prostate cancer. It is worth noting that we treated the mice with 0.50 mg/kg chetomin, with no significant weight loss observed, in comparison to the 1–2 mg/kg chetomin that induced toxicity in a previous study [23]. We also measured the extent of necrosis in tumor sections stained with H & E, and found a statistically significant increase in necrosis in tumors treated with gliotoxin compared to vehicle, chaetocin- and chetomin-treated tumors. This is consistent with the fact that under hypoxic conditions, oxidative phosphorylation is impaired leading to the induction of glycolytic enzymes, which maintain the basal level of adenosine 5’-triphosphate required for cell survival [36]. Therefore, inhibition of glycolytic genes, such as *LDHA* and *ENO1*, by ETPs most likely played a role in inhibiting cell survival under hypoxia and promoting cell death in hypoxic areas. Thus, although the ETPs exhibited antiangiogenic effects *in vitro,* their mechanism of action *in vivo*, at least at the dose tested, appears to be via direct cytotoxic effects on tumor cells. This may explain why VEGF and CD31 did not reveal appreciable differences between vehicle and ETP treatment groups, although as noted earlier, definitive conclusions could not be made since the immunostaining data were difficult to interpret.

In conclusion, although a number of anticancer drugs have been shown to inhibit HIF, none of these drugs have been shown to function as direct inhibitors. In addition, most HIF-1 inhibitors that have been put forth have failed to demonstrate therapeutic efficacy in clinical trials for cancer patients, and these failures have been due, at least in part, to their lack of specificity. Thus, there has been a clear need for novel, potent, inhibitors that target clearly defined points in the HIF pathway. Although future studies are needed to characterize the specificity of ETPs, particularly with respect to other zinc-binding proteins, the results presented suggest that directly targeting the HIF-1α/p300 complex with ETPs may be an effective approach for inhibiting angiogenesis and tumor growth. While the usefulness of ETPs such as chetomin or gliotoxin is limited by their toxicity, there specificity in targeting HIF-1 makes them attractive molecules for the design of future chemotherapeutic agents.

## Competing interests

The authors declare that they have no competing interests.

## Authors’ contributions

KR conceived the study and designed the experiments, performed the molecular experiments, and drafted the manuscript. ER and TC performed the molecular experiments. KC performed the molecular experiments and helped with experimental design. SP and AH carried out the animal experiments. DJ and DL performed the statistical analyses. CC and DP critically revised the manuscript for intellectual content. WF supervised the study. All authors read and approved the final manuscript.

## Supplementary Material

Additional file 1: Figure S1 ETPs decrease VEGF secretion in HCT116 cells. *A,* Hypoxia was induced for 18 h in HCT116 cells in the absence or presence of the indicated concentrations of ETPs, followed by ELISA quantification of secreted VEGF normalized to DMSO under hypoxic conditions (*n* = 1-6). A repeated measures ANOVA was performed on the data; Hochberg’s method was used to adjust the *p*-values. *, *p* < 0.05, **, *p* < 0.001, ***, *p* < 0.0001. *B,* Cell viability was determined in HCT116 cells using the CellTiter-Blue cell viability reagent. Data points are presented as mean ± S.E.M from independent experiments (*n*=3-7). Click here for file

Additional file 2: Figure S2 ETPs decrease expression of HIF-1α-dependent target genes in HCT116 cells. Cells were incubated in the absence or presence of the indicated concentrations of gliotoxin (*top panel*), chaetocin (*middle panel*), and chetomin (*bottom panel*). Total RNA was harvested and tested for *VEGF*, *LDHA*, and *ENO1* mRNA expression by qPCR. Results are expressed as fold increase relative to mRNA levels under normoxic conditions in the absence of ETPs. β-actin was tested in parallel as an internal control for input RNA. Results are the mean ± S.E.M of independent experiments run (*n*=2-6). A repeated measures ANOVA was performed on the data; Hochberg’s method was used to adjust the *p-*values *, *p* < 0.05, **, *p* < 0.001, ***, *p* < 0.0001. Click here for file
