# Supplementary figures and images for: Epidithiodiketopiperazines (ETPs) exhibit in vitro antiangiogenic and in vivo antitumor activity by disrupting the HIF-1α/p300 complex in a preclinical model of prostate cancer
Source: Mol Cancer. 2014 Apr 28;13:91. doi: 10.1186/1476-4598-13-91 (PMC4113146; doi:10.1186/1476-4598-13-91)

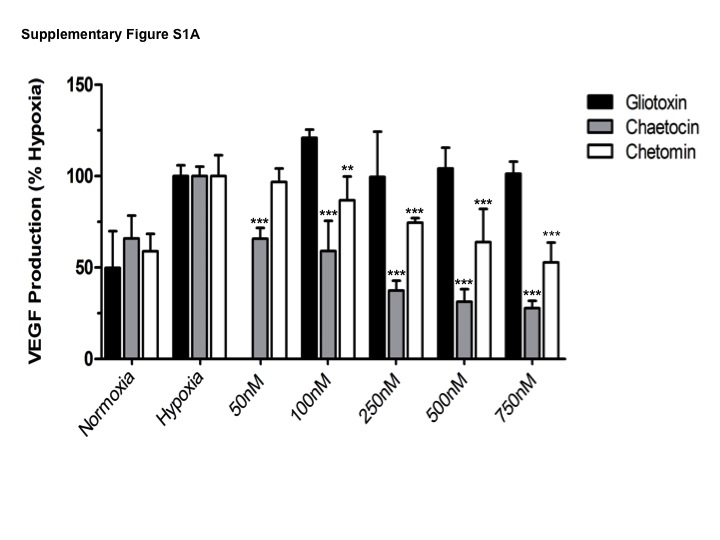

Supplement: Additional file 1: Figure S1 — ETPs decrease VEGF secretion in HCT116 cells. A, Hypoxia was induced for 18 h in HCT116 cells in the absence or presence of the indicated concentrations of ETPs, followed by ELISA quantification of secreted VEGF normalized to DMSO under hypoxic conditions (n = 1-6). A repeated measures ANOVA was performed on the data; Hochberg’s method was used to adjust the p-values. *, p < 0.05, **, p < 0.001, ***, p < 0.0001. B, Cell viability was determined in HCT116 cells using the CellTiter-Blue cell viability reagent. Data points are presented as mean ± S.E.M from independent experiments (n=3-7). [file 1476-4598-13-91-S1.zip › 3414585561046591_add1/3414585561046591_add1a.jpeg]

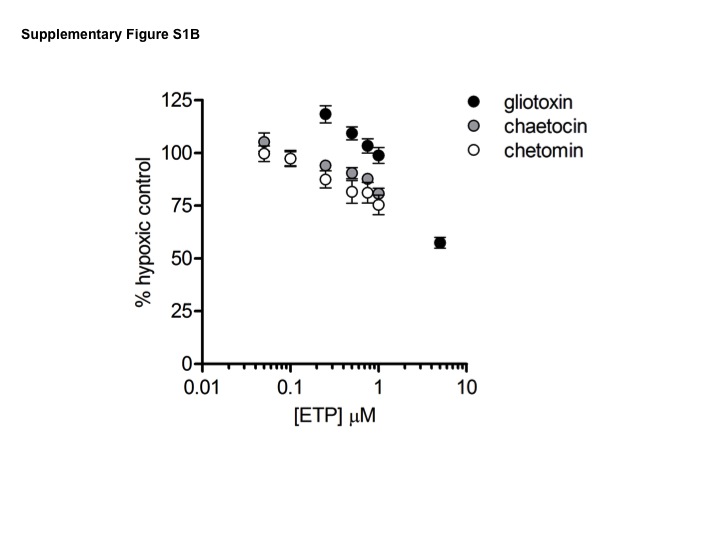

Supplement: Additional file 1: Figure S1 — ETPs decrease VEGF secretion in HCT116 cells. A, Hypoxia was induced for 18 h in HCT116 cells in the absence or presence of the indicated concentrations of ETPs, followed by ELISA quantification of secreted VEGF normalized to DMSO under hypoxic conditions (n = 1-6). A repeated measures ANOVA was performed on the data; Hochberg’s method was used to adjust the p-values. *, p < 0.05, **, p < 0.001, ***, p < 0.0001. B, Cell viability was determined in HCT116 cells using the CellTiter-Blue cell viability reagent. Data points are presented as mean ± S.E.M from independent experiments (n=3-7). [file 1476-4598-13-91-S1.zip › 3414585561046591_add1/3414585561046591_add1b.jpeg]

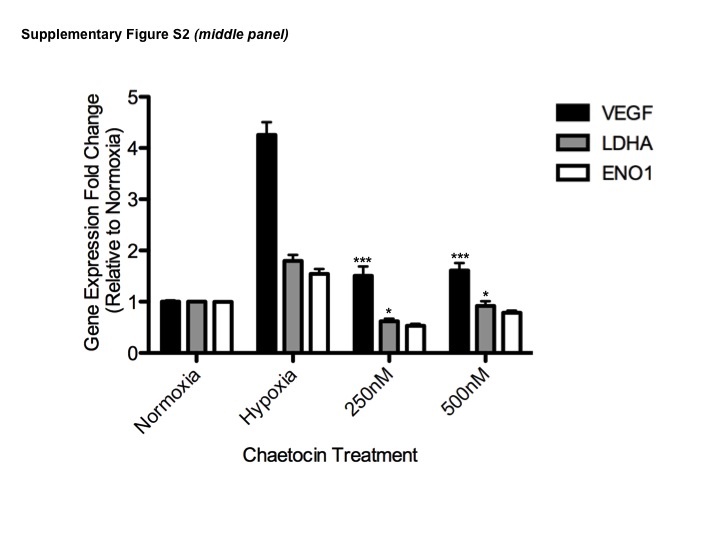

Supplement: Additional file 2: Figure S2 — ETPs decrease expression of HIF-1α-dependent target genes in HCT116 cells. Cells were incubated in the absence or presence of the indicated concentrations of gliotoxin (top panel), chaetocin (middle panel), and chetomin (bottom panel). Total RNA was harvested and tested for VEGF, LDHA, and ENO1 mRNA expression by qPCR. Results are expressed as fold increase relative to mRNA levels under normoxic conditions in the absence of ETPs. β-actin was tested in parallel as an internal control for input RNA. Results are the mean ± S.E.M of independent experiments run (n=2-6). A repeated measures ANOVA was performed on the data; Hochberg’s method was used to adjust the p-values *, p < 0.05, **, p < 0.001, ***, p < 0.0001. [file 1476-4598-13-91-S2.zip › 3414585561046591_add2/Slide18.jpg]

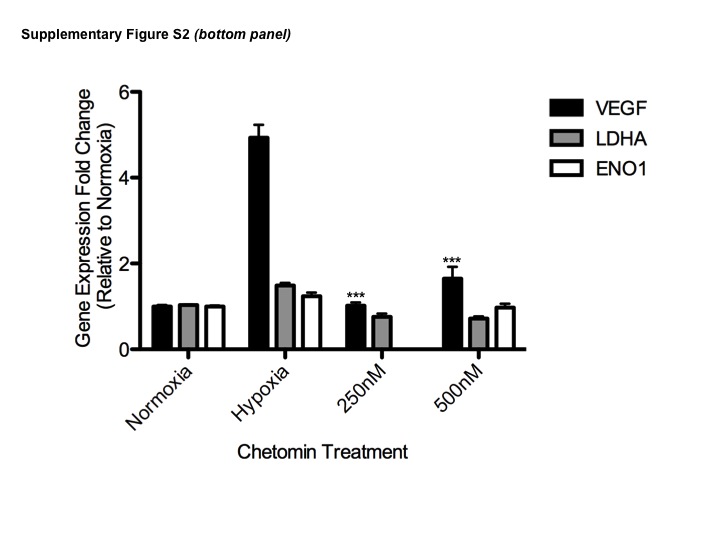

Supplement: Additional file 2: Figure S2 — ETPs decrease expression of HIF-1α-dependent target genes in HCT116 cells. Cells were incubated in the absence or presence of the indicated concentrations of gliotoxin (top panel), chaetocin (middle panel), and chetomin (bottom panel). Total RNA was harvested and tested for VEGF, LDHA, and ENO1 mRNA expression by qPCR. Results are expressed as fold increase relative to mRNA levels under normoxic conditions in the absence of ETPs. β-actin was tested in parallel as an internal control for input RNA. Results are the mean ± S.E.M of independent experiments run (n=2-6). A repeated measures ANOVA was performed on the data; Hochberg’s method was used to adjust the p-values *, p < 0.05, **, p < 0.001, ***, p < 0.0001. [file 1476-4598-13-91-S2.zip › 3414585561046591_add2/Slide19.jpg]
